# WDR62 Regulates Early Neural and Glial Progenitor Specification of Human Pluripotent Stem Cells

**DOI:** 10.1155/2017/7848932

**Published:** 2017-06-13

**Authors:** Abdullah J. Alshawaf, Ana Antonic, Efstratios Skafidas, Dominic Chi-Hung Ng, Mirella Dottori

**Affiliations:** ^1^Centre for Neural Engineering, The University of Melbourne, Carlton, VIC 3010, Australia; ^2^Department of Psychiatry, The University of Melbourne, Carlton, VIC 3010, Australia; ^3^Department of Electrical and Electronic Engineering, The University of Melbourne, Carlton, VIC 3010, Australia; ^4^Department of Medicine, The University of Melbourne, Royal Melbourne Hospital, Parkville, VIC, Australia; ^5^School of Biomedical Science, Faculty of Medicine and Biomedical Sciences, University of Queensland, St Lucia, QLD 4072, Australia; ^6^Department of Anatomy and Neuroscience, The University of Melbourne, Carlton, VIC 3010, Australia

## Abstract

Mutations in WD40-repeat protein 62 (*WDR62*) are commonly associated with primary microcephaly and other developmental cortical malformations. We used human pluripotent stem cells (hPSC) to examine WDR62 function during human neural differentiation and model early stages of human corticogenesis. Neurospheres lacking WDR62 expression showed decreased expression of intermediate progenitor marker, TBR2, and also glial marker, S100*β*. In contrast, inhibition of c-Jun N-terminal kinase (JNK) signalling during hPSC neural differentiation induced upregulation of WDR62 with a corresponding increase in neural and glial progenitor markers, PAX6 and EAAT1, respectively. These findings may signify a role of WDR62 in specifying intermediate neural and glial progenitors during human pluripotent stem cell differentiation.

## 1. Introduction

WD40-repeat protein 62 (WDR62) mutations are common genetic causes of human autosomal recessive primary microcephaly (MCPH), a neurodevelopmental disorder characterized by significantly reduced cerebral cortex size at birth [1]. WDR62 mutations have also been implicated in several other cortical malformations, suggesting pleiotropic functions during brain development [2, 3]. Wdr62 is a microtubule-associated scaffold protein that is regulated by tiered c-Jun N-terminal kinase (JNK) signalling modules [4, 5]. Wdr62 studies involving embryonic mouse brains and human cell lines demonstrate its dynamic cell cycle-dependent spindle pole localization and mitotic function [3, 6]. Knockdown of Wdr62 expression in rat embryos disturbs cell division of neural progenitors in the ventricular (VZ) and subventricular (SVZ) zones, resulting in decreased proliferation and premature differentiation [4]. Other studies examining Wdr62 mutant mice attributed the reduction of neural progenitors to increased cell death resulting from cell cycle arrest rather than altered differentiation of progenitors [7]. Taken together, these studies support a role for WDR62 in neurogenesis during brain development although the precise role and range of WDR62 functions remain unclear [2–4, 8, 9].

While studies in mice and immortalized cell lines have greatly contributed to our understanding of WDR62's role in neurogenesis, it is important to further investigate its function within the context of human model systems. Human pluripotent stem cells (hPSC) may be utilised to model processes associated with cortical development and related neurodevelopmental disorders in vitro [10, 11]. Accordingly, here, we investigated the role of WDR62 in specification to neural and astrocyte progenitors via using an inducible WDR62-depleted hPSC line and implementation of well-established protocols that generate cortical neural and glial progenitors. Our findings show that downregulation of WDR62 leads to reduction in intermediate neural and glial progenitors derived from hPSC. Consistent with these findings, specific inhibition of JNK signalling in hPSC-derived neural cultures enhanced expression of WDR62, giving rise to an increase in neural and glial progenitors. Taken together, these findings suggest that WDR62 is required for specification of early neural and glial progenitors and a role for JNK signalling in negatively regulating WDR62 in hPSC.

## 2. Materials and Methods

### 2.1. Maintenance of hPSC and Generation of shRNA Knockdown Lines

This project is approved by the University of Melbourne Human Ethics committee (number 1545384). The H9 (WA-09, WiCell) cell line was a cultured feeder free on vitronectin-coated plates using MTeSR-1 defined media according to the manufacturer's instructions (Stem Cell Technologies) and maintained at 37°C 5% CO2. Colonies were mechanically dissected every 7 days and transferred to freshly coated plates. Cell culture media were changed every day.

shRNA knockdown was performed using SMARTchoice inducible lentiviral small hairpin RNA (shRNA) targeting human WDR62 (H15) (Dharmacon, catalog number: VSH6376). H9 cells were plated on organ culture dish and were transduced as per manufacturer's instructions at MOI of 0.5 and selected using 0.5 *μ*g/mL puromycin. At day 7, transduced hPSC (hPSC-H15) were temporarily treated with 1 *μ*g/ml doxycycline (Dox) to induce expression of GFP and further perform manual selection.

### 2.2. Neural and Glial Differentiation

Neural induction of H9 cells and/or WDR62 shRNA-transduced H9 cells were set up as described by Denham and Dottori, with some slight modifications [12]. hPSC and/or hPSC-H15 were mechanically dissected into pieces approximately 0.5 mm in width and transferred to laminin-coated organ culture plates in N2B27 medium containing 1 : 1 mix of neurobasal medium with DMEM/F12 medium, supplemented with 1% insulin/transferrin/selenium, 1% N2, 1% retinol-free B27, 0.3% glucose, 25 U/ml penicillin, and 25 *μ*g/ml streptomycin (Life Technologies/Invitrogen). For differentiation to cortical neurons, the small molecule inhibitor SB431542 (10 *μ*M, Tocris) and noggin (500 ng/ml, Peprotech) were added to the media for the first 7 days followed by the addition of fibroblast growth factor (FGF) (20 ng/ml, Peprotech) for the remaining 7 days. Following 2 weeks of neural induction, neural progenitors were mechanically harvested and cultured in suspension in neural basal media (NBM) supplemented with FGF (20 ng/ml, Peprotech) and epidermal growth factor (EGF) (20 ng/ml, Peprotech) to promote neurosphere (NSP) formation [13]. To differentiate to glial cells, pieces of neural induction colonies were plated onto fibronectin-coated organ culture plates containing NBM supplemented with FGF (20 ng/ml, Peprotech), EGF (20 ng/ml, Peprotech), and PDGF-AA (20 ng/ml, Peprotech) for one week followed by an additional week without supplements. shRNA-transduced hPSC cells were constantly treated with 0.5 *μ*g/mL puromycin. Dox treatment commenced from the beginning of the induction with no Dox controls set up in parallel. Media change was performed every second day. At the end of the experiment, samples were either fixed and prepared for immunostaining or processed for real-time quantitative PCR (Q-PCR) expression analyses.

### 2.3. Immunostaining

Cell monolayers and NSPs were fixed in 4% PFA for 20 minutes at 4°C and then washed briefly in PBS. NSPs were embedded in Tissue-Tek OCT compound (Labtek) and cut at 15 *μ*m on a cryostat, and sections were placed on superfrost slides. After washing in PBS, sections or culture dishes were permeabilized with 0.1% Triton-X-100 in PBS (PBT) for 5 minutes and then blocked in 10% fetal calf serum in PBT for 60 minutes at room temperature. Samples were then incubated with primary antibody (diluted in the block buffer) overnight at 4°C. The following primary antibodies were used: goat anti-SOX2 (1 : 200, R&D Systems), mouse anti-PAX6 (1 : 80, DSHB), rabbit anti-TBR2 (1 : 1000, Chemicon), mouse anti-TUJ1 (1 : 1000, Millipore), rabbit anti-KI67 (1 : 600, Abcam), and mouse anti-S100*β* (1 : 500, Sigma). Following three 5 minute washes in PBT, ALEXA Fluor secondary antibodies (Life Technologies/Invitrogen) (1 : 1000 diluted in the block buffer) were applied for 1 hour at room temperature. All samples were counterstained with 49,6-diamidino-2-phenylindole (DAPI; 1 *μ*g/ml, Sigma-Aldrich). Samples were then mounted onto glass slides with 5 *μ*l of moviol aqueous mountant followed by viewing and image capturing under Zeiss Axio Observer z1 fluorescence microscope using ZEN imaging software.

### 2.4. Gene Expression Analysis

Cells were harvested and processed for total RNA extraction using PureLink RNA Mini Kit (Life Technologies). Quality of RNA was examined on NanoDrop 2000 Spectrophotometer (Thermo Scientific) with A_260/280_ ratio ranging from 1.95 to 2.05. Up to 2 *μ*g of RNA was used to synthesize first-strand cDNA using SensiFAST cDNA Synthesis Kit (Bioline). Q-PCR reaction and data collection was performed on ViiA7 Real-Time PCR System (Life Technologies) using Universal Master Mix (Applied Biosystems), and raw data were exported to Microsoft Excel for further analysis. Cycling parameters were as follows: 50°C for 2 minutes, 95°C for 10 minutes to activate DNA polymerase, then 40 cycles of 95°C for 15 seconds, and 60°C for 1 minute. ΔCT values were obtained by normalization to the mean of three internal reference genes GAPDH, HMBS, and ELF1. Next, ΔΔCT values were obtained by normalization to ΔCT values of appropriate control samples. Relative gene expression values (fold change) were then calculated using the 2^−∆∆CT^ method [14]. Statistical significance tests were performed using unpaired *t*-test and one-way ANOVA with post hoc Bonferroni multiple comparisons.

### 2.5. NSP Size Measurements

To compare the size of hPSC-H15-derived NSPs treated with Dox versus no Dox treatment, for each independent experiment (*n* = 4), brightfield images were randomly taken for at least 3 NSPs for each condition using Zeiss Axio Observer z1 fluorescence microscope and ZEN imaging software. All images were taken at a consistent magnification for each experiment. ImageJ software was used to estimate the largest diameter of each NSP, briefly, by manually drawing a straight line across the centre of the NSP and the length of which was documented in *μ*m. Statistical significance tests were performed using Mann–Whitney test.

### 2.6. Quantification of S100*β*

WDR62 shRNA-transduced hPSC underwent glial differentiation for 2 weeks and then processed for immunofluorescence staining of S100*β* as described above. S100*β*-positive neurons in Dox treated and no Dox treatment were analysed from at least 3 replicates. Images for analysis were taken using 20x objective Zeiss Axio Observer z1 fluorescence microscope and ZEN imaging software. ImageJ software was used to quantify integrated density of S100*β* staining within images for each replicate. The results from Dox treated were compared to the normalized results obtained from the no Dox group (set at 100%). Two-tailed *t*-test was used to determine statistical significance.

### 2.7. JNK Inhibition

JNK inhibitor was obtained from EMD Millipore (catalog number: 420135), reconstituted in dimethyl sulfoxide (DMSO) to 50 mM, and used at 50 *μ*M. H9 neural inductions were treated with JNK inhibitor from day 0 of neural induction, and DMSO-treated control was always set up in parallel. The experiment was conducted at least three times. Cultures were harvested for Q-PCR analyses. Two-tailed *t*-test was used to determine statistical significance.

## 3. Results

### 3.1. WDR62 Is Expressed during Neural Induction Coinciding with Early Neural Progenitors

**Figure 1:**
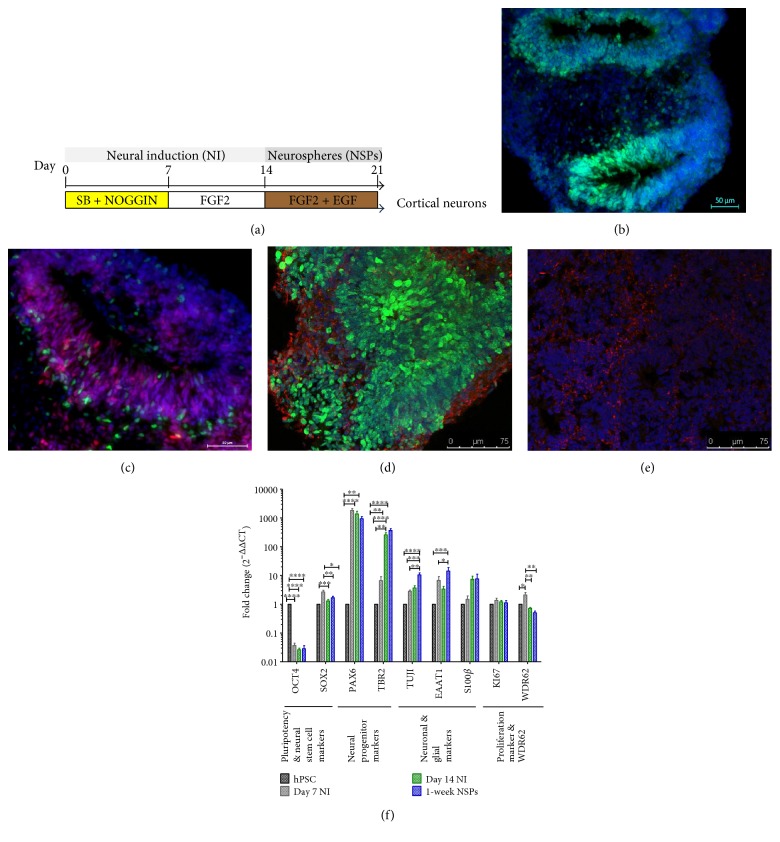
Expression of WDR62 in hPSC-derived neural progenitors. (a) Schematic diagram outlining neural induction protocol for deriving cortical neuronal progenitors. (b-c) DS neural induction protocol generates neural progenitors expressing early neural progenitor markers SOX2 (b, green) and PAX6 (c, red) and intermediate progenitor marker TBR2 (c, green). DAPI stains of nuclei are also shown. Scale bars = 50 *μ*m. (d-e) hPSC-derived 1-week-old NSPs also express neuronal marker TUJ1 (d, red), proliferative marker KI67 (d, green), and glial marker S100*β* (e, red). DAPI stains of nuclei are shown in (e). Scale bars = 75 *μ*m. (f) Gene expression analyses of pluripotency marker OCT4; neural progenitor markers SOX2, PAX6, and TBR2; neuronal marker TUJ; glial markers EAAT1 and S100*β*; and proliferative marker KI67 and WDR62 across several time points of the neural induction protocol, including hPSC, day 7 NI, day 14 NI, and 1-week NSPs (*n* ≥ 4 independent experiments for each time point, >3 NSPs per experiment). Data presented as means ± S.E.M. one-way ANOVA with a post hoc Bonferroni multiple comparison was used to assess significance for each gene, comparing all pairs of time points. ^∗^*P* < 0.05; ^∗∗^*P* < 5.0*E* − 03; ^∗∗∗^*P* < 1.0*E* − 04; ^∗∗∗∗^*P* < 1.0*E* − 05. NI: neural induction; NSPs: neurospheres.

To determine whether WDR62 was upregulated during hPSC neural differentiation, endogenous WDR62 expression was measured by Q-PCR during stages of hPSC neural induction and differentiation to neural and glial progenitors. The protocol used for neural induction was the dual SMAD inhibition which involves treating hPSC with inhibitors of the bone morphogenic protein and/or activin/nodal signalling pathways, noggin, and SB431542, respectively, for 7 days followed by FGF for another 7 days [15]. hPSC-derived progenitors were organize in a rosette-like morphology expressing early telencephalic progenitor marker PAX6 and intermediate progenitor marker TBR2 (Figure 1). Neural progenitors are mechanically harvested and cultured in a suspension supplemented with FGF and EGF for one week to form NSP aggregates. NSPs cultured for one week consisted of mixed populations of early intermediate and postmitotic neural and glial progenitors as indicated by expression of SOX2, PAX6, TBR2, TUJ1, and S100*β* (Figure 1) [16, 17].

WDR62 expression was significantly upregulated during early stages of neural induction at day 7 (ANOVA, *P* < 0.05, *n* = 5), which co-coincided with the onset of SOX2 and PAX6 expression (Figure 1(f)). WDR62 expression was then significantly reduced at the end of neural induction (ANOVA, *P* < 5.0*E* − 03, *n* = 5) and also at the NSP stage (ANOVA, *P* < 5.0*E* − 03, *n* = 5), which corresponded with an increase in expression of TBR2, TUJ1, EAAT1, and S100*β* (Figure 1(f)). These findings suggest that WDR62 is upregulated at the onset of hPSC neural induction, correlating with specification of early neural progenitors.

### 3.2. Inducible Knockdown of WDR62 Expression during hPSC Neural Induction

**Figure 2:**
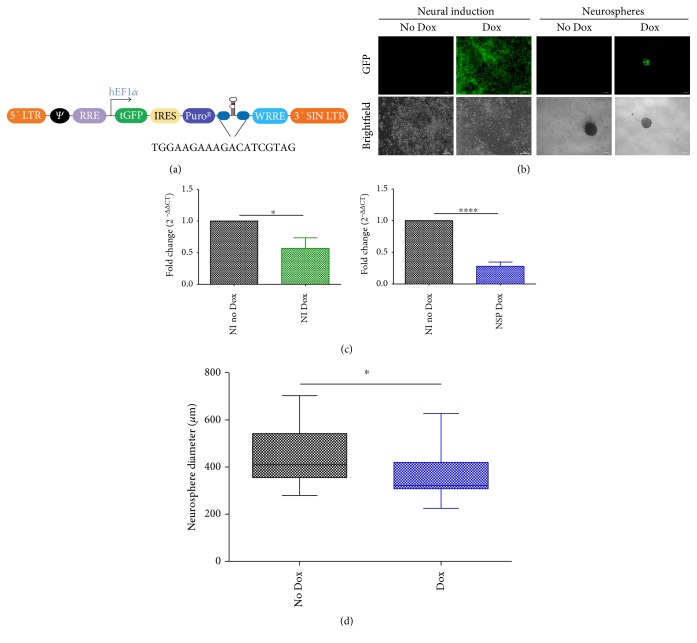
Efficient knockdown of WDR62 expression in hPSC-derived neural progenitors. (a) Map of inducible lentiviral shRNA vector and targeted sequence of WDR62. (b) GFP and corresponding brightfield images of WDR62 shRNA-transduced hPSC-derived neural progenitors with versus without Dox treatment at neural induction and NSP stages. (c) Gene expression analyses showing downregulation of WDR62 expression in WDR62 shRNA-transduced hPSC-derived neural progenitors treated with Dox versus no Dox treatment at neural induction (*P* = 0.04) and NSP stages (*P* = 7.3*E* − 6) (*n* = 4 and *n* = 6 independent experiments, resp., >3 NSPs per experiment). Two-tailed *t*-test was used to assess significance. (d) Diameter measurements of WDR62 shRNA-transduced hPSC-derived NSPs treated with Dox versus no Dox controls (*n* = 3 independent experiments, >3 NSPs per experiment). Mann–Whitney *U* test was used to assess significance. ^∗^*P* < 0.05; ^∗∗∗∗^*P* < 5.0*E* − 05. NI: neural induction; NSPs: neurospheres; Dox: doxycycline.

Given that WDR62 expression was upregulated at the early stages of neural induction, we explored the impact of reduced WDR62 expression on hPSC neural differentiation. For these analyses, hPSC were transduced with WDR62 Dox-inducible lentiviral shRNA construct (H15), which coexpress GFP. hPSC-H15 underwent neural induction and differentiation using the dual SMAD inhibition protocol, as previously described, either in the presence or absence of doxycycline (Dox/no Dox) (Figure 2). Treatment with Dox efficiently induced universal GFP expression in hPSC-H15-derived neural progenitors at the neural induction stage, and GFP expression was maintained in 1-week-old NSPs (Figure 2(b)). At the neural induction stage, Dox-treated hPSC-H15 exhibited significant mean reduction (40%) in the expression of WDR62 relative to no Dox treatment (two-tailed *t*-test, *P* = 0.04, *n* = 4) (Figure 2(c)). Similarly at the NSP stage, WDR62 expression was reduced by more than 70% in Dox-treated hPSC-H15-derived NSPs compared to no Dox-treated NSPs (two-tailed *t*-test, *P* = 7.3*E* − 6, *n* = 6) (Figure 2(c)). Although NSPs were generated in conditions with reduced WDR62 expression, their size was significantly smaller than the no Dox-treated controls (Mann–Whitney *U* test, *P* = 0.03) (Figure 2(d), Supplemental Table 1 available online at https://doi.org/10.1155/2017/7848932). Overall, these results confirm that the Dox induction of WDR62 shRNA was effective at reducing endogenous WDR62 expression in hPSC-derived neural stem cells and progenitors.

### 3.3. Downregulation of WDR62 during Neural Differentiation Reduced Early Neural and Glial Progenitors

**Figure 3:**
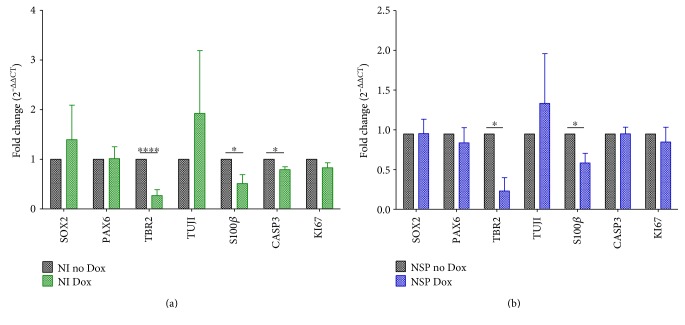
WDR62 knockdown effects hPSC specification to neural and glial progenitors. (a) Q-PCR data suggest significant reduction of TBR2 (*P* = 8.0*E* − 04), S100*β* (*P* = 0.03), and CASP3 (*P* = 0.02) expression in Dox-treated WDR62 shRNA-transduced hPSC-derived neural progenitors treated with Dox versus no treatment at day 14 neural induction (*N* ≥ 3 independent experiments). Two-tailed *t*-test was used to assess significance. (b) Similarly, Q-PCR data suggest significant reduction of TBR2 (*P* = 0.01) and S100*β* (*P* = 0.01) expression in Dox-treated versus no Dox treatment at the NSP stage (*N* ≥ 3 independent experiments, >3 NSPs per experiment). Two-tailed *t*-test was used to assess significance. ^∗^*P* < 0.05; ^∗∗∗∗^*P* < 5.0*E* − 05. NI: neural induction; NSPs: neurospheres; Dox: doxycycline.

To investigate further the observed effect of WDR62 depletion resulting in NSP size reduction, we analysed the expression of neural stem cell and progenitor markers in Dox-treated hPSC-H15 cultures by Q-PCR at both the neural induction and NSP stages of differentiation. We did not observe significant changes in gene expression of proliferation marker KI67 or cell death marker caspase 3 (CASP3) with reduced WDR62 expression (Figures 3(a) and 3(b)). Expression of mitotically active neural progenitor markers, SOX2 and PAX6, was also not significantly changed (Figures 3(a) and 3(b)). In contrast, expression of TBR2, marker of intermediate neural progenitors, and the glial marker S100*β* was both significantly reduced in neural induction cultures (two-tailed test, *P* = 8.0*E* − 04 for TBR2 and *P* = 0.03 for S100*β*, *n* ≥ 3) and NSPs (two-tailed *t*-test, *P* = 0.01 for TBR2 and *P* = 0.01 for S100*β*, *n* ≥ 3) following Dox-induced WDR62 knockdown (Figures 3(a) and 3(b)). Interestingly, reduced TBR2 and WDR62 expression in NSPs corresponded with a trend towards increased TUJ1 expression, a marker of postmitotic neurons (Figure 3(b)). Taken together, these results suggest a role of WDR62 in hPSC neural differentiation, particularly in hPSC specification to early neural and glial progenitors.

### 3.4. WDR62 Is Required for Differentiation to Glial Populations

**Figure 4:**
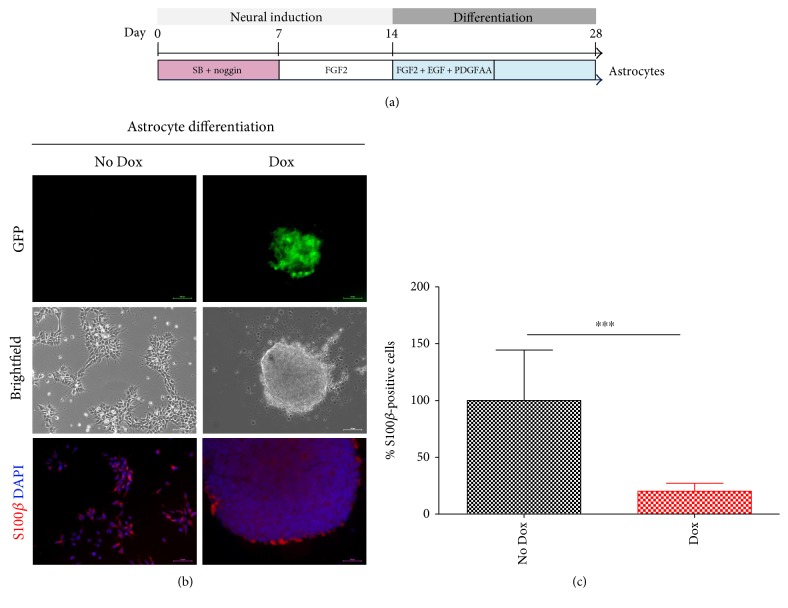
WDR62 knockdown effects differentiation to astrocytes. (a) Schematic diagram outlining protocol for hPSC differentiation to astrocytes. (b) GFP, brightfield, and S100*β* immunostaining images of WDR62 shRNA-transduced hPSC-derived astrocytes with versus without Dox treatment at the end of differentiation. DAPI stains of nuclei are shown. Scale bars = 50 *μ*m. (c) Quantification analyses of S100*β-*positive cells suggesting significantly decreased (*P* = 4.9*E* − 04) differentiation to astrocytes in Dox-treated group versus no Dox treatment controls (*n* = 3 independent experiments). Two-tailed *t*-test was used to assess significance. ^∗∗∗^*P* < 5.0*E* − 04. Dox: doxycycline.

To further determine whether reduced WDR62 expression impacted on the differentiation of hPSC to glial progenitors, hPSC-H15 was differentiated to glia in both Dox and no Dox treatment conditions. The glial differentiation protocol is similar to the neural induction protocol described above; however after 14 days cells are passaged onto fibronectin substrate in media supplemented with FGF, EGF, and PDGF-AA for one week followed by media without supplements for another week (Figure 4(a)). It was found that Dox-treated glial differentiation cultures, which had reduced WDR62 expression, resulted in a significant decrease in the number of S100*β-*positive cells compared to controls (two-tailed *t*-test, *P* = 4.9*E* − 04, *n* = 3) (Figure 4). This finding further supports the requirement of WDR62 for early stages of hPSC glial differentiation.

### 3.5. Inhibition of JNK Enhances the Expression of WDR62 and PAX6

**Figure 5:**
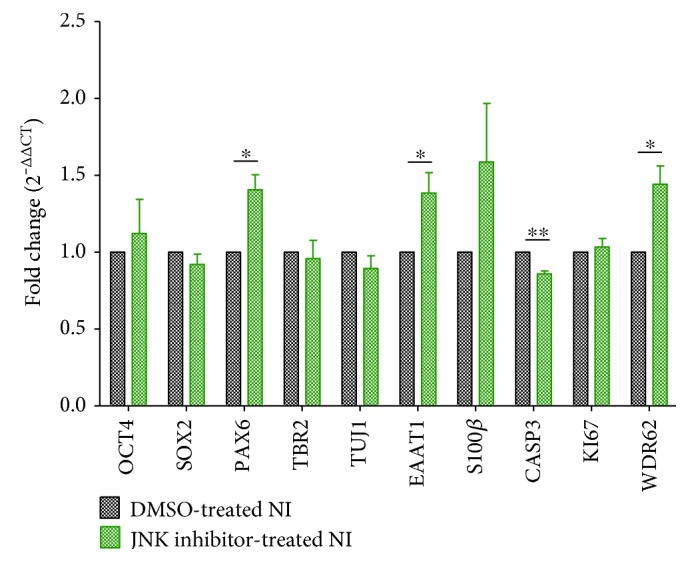
JNK inhibition effect on the expression of WDR62 and hPSC specification to neural and glial progenitors. Q-PCR data shows significant expression increase of PAX6 (*P* = 0.01), EAAT1 (*P* = 0.04), and WDR62 (*P* = 0.02) and decreased expression of CASP3 (*P* = 1.0*E* − 03) in hPSC-derived neural progenitors treated with JNK inhibitor versus no treatment at day 14 neural induction (*n* = 3 independent experiments). Two-tailed *t*-test was used to assess significance. ^∗^*P* < 0.05; ^∗∗^*P* < 5.0*E* − 03.

To further investigate the mechanisms underlying WDR62 function, we examined the contribution of JNK activity as WDR62/JNK signalling interactions were previously shown to play a critical role in cortical development [4, 18]. Thus, we attenuated JNK activity in our differentiation system with a kinase-specific chemical inhibitor (JNK inhibitor VIII, 50 mM) at doses previously demonstrated to attenuated WDR62/JNK signalling to downstream substrate [6]. We then assessed the expression of WDR62 and candidate genes related to neural and glial differentiation, cell death, and proliferation by Q-PCR. Our results indicate significantly enhanced expression of WDR62 (two-tailed *t*-test, *P* = 0.02, *n* = 3) following JNK inhibition, compared to DMSO-treated control samples (Figure 5). This was accompanied by significantly increased expression of PAX6 (two-tailed *t*-test, *P* = 0.01, *n* = 3) and functional glial marker EAAT1 (two-tailed *t*-test, *P* = 0.04, *n* = 3) (Figure 5). In addition, there was a trend towards increased S100*β* following JNK inhibition. These findings are consistent with reports of JNK being a negative regulator of WDR62 mitotic function and expression and are consistent with the role of WDR62 in neuro- and gliogenesis [6].

## 4. Discussion

This study is the first to utilise hPSC-based modelling system to investigate the role of WDR62 in neurogenesis. We show that WDR62 is expressed in hPSC-derived neural progenitors with the highest expression occurring early in neural induction coinciding with the onset of early neural progenitor markers SOX2 and PAX6. This is consistent with previous reports describing Wdr62 expression in precursors undergoing mitosis in the VZ, SVZ, and cortical plate [4, 8]. Others have also reported expression of WDR62 in postmitotic neurons [7]. Here, we show that knockdown of WDR62 expression in hPSC-derived neural cultures resulted in altered differentiation to early neural and glial progenitors as indicated by the reduced expression of TBR2 and S100*β*. Related to these findings, previous studies in Wdr62 mutant mice demonstrated cortical defects whereby PAX6 and Tbr2 expression were significantly reduced within the cortical regions; however, there were no significant differences in Tbr2 expression specifically within the VZ regions [4, 7]. Accompanied with this depletion of neural progenitors, one study observed reduced cell proliferation and increased premature neuronal differentiation while another study reported mitotic delay and increased cell death [4, 7]. In contrast to these findings, Jayaraman et al. found an increased ratio of TBR2^+^/SOX2^+^ progenitors and increased Ki67^+^ PAX6^−^ cells beyond VZ and SVZs in embryonic day 14 Wdr62 null mice and reduced overall cortical thickness with no evidence of increased cell death. This led the authors to conclude that loss of Wdr62 causes cell fate change specifically in switching from PAX6 to Tbr2 progenitors with no cell death, resulting in premature delamination of PAX6 progenitors from VZ [19]. In our studies, we did not observe any differences in markers of cellular proliferation or cell death in WDR62 knockdown hPSC cultures, although NSP size were smaller, suggesting that only subpopulations of progenitors were affected by the downregulation of WDR62. In light of these findings, we propose that WDR62 is likely to play a critical role in the specification of early neural and glial progenitors.

During cortical development, PAX6 expressing radial glial cells residing within the VZ undergo self-renewal and/or differentiation to give rise to intermediate neural/glial progenitors, postmitotic cortical neurons, or glia [20–23]. Intermediate neural progenitors are identified by the expression of Tbr2, which divide and differentiate within the SVZ to produce more neurons [20–22, 24–26]. Similar to cortical neurons, astrocytes are produced either directly from radial glia or indirectly from more glial restricted intermediate progenitors within the SVZ [27, 28]. Towards the end of cortical development, the majority of radial glial cells lose their identity to differentiate to astrocytes expressing S100*β* [27, 29, 30]. Given this knowledge, it may be that WDR62 plays a role in transitioning hPSC-derived PAX6 neural precursors to intermediate progenitors of neural and glial lineages. Similar to radial glial cells, there may be hPSC-derived PAX6 precursor subpopulations that bypass the intermediate TBR2 progenitor stage and directly differentiate to postmitotic neurons, which may explain why there were no significant changes in TUJ1 expression in the WDR62 knockdown NSPs. Alternatively, or in addition to, WDR62 may play a role in self-renewal of intermediate neural/glial progenitors, but not in self-renewal of PAX6 neural precursors. Further in-depth studies of WDR62 expression and function are needed to determine the underlying mechanisms of how WDR62 regulates hPSC neural differentiation. Overall, the findings from this study shed light on WDR62's role in human corticogenesis.

The WDR62 mitotic function has been linked to the activity of JNK [6, 9]. It has been shown that WDR62 recruits JNK1 to the spindle microtubules specifically during mitosis and reciprocally JNK1 regulates WDR62 intercellular distribution. Specifically, JNK phosphorylation of WDR62 negatively regulates the association of WDR62 with the spindle microtubules [6]. As a result of inhibiting JNK signalling, it has been shown that the WDR62 microtubules association was persistent irrespective of the cell cycle stage [6]. Our results indicate that JNK activity is not required for WDR62 function with regard to specifying neural and glial progenitors derived from hPSC. In contrast, treatment with JNK-specific inhibitor increased WDR62 expression in hPSC-derived neural progenitors and this coincided with enhanced expression of PAX6-positive cells and astrocyte functional marker EAAT1. Thus, our findings indicate that JNK activity may also negatively regulate the expression levels of WDR62. Nevertheless, our studies in hPSC-derived neural progenitors suggest that WDR62 expression is required for the specification of specific neural and glial lineages and this does not appear to require JNK signalling activity, a finding that is contrary to rodent studies.

## 5. Conclusions

This study demonstrates the robustness of hPSC as a complementary modelling system investigating the developmental roles of candidate genes implicated in major brain developmental disorders. Our findings show that downregulation of WDR62 leads to reduction in intermediate neural and glial progenitors derived from hPSC. Consistent with these findings, specific inhibition of JNK in hPSC-derived neural cultures enhanced the expression WDR62, giving rise to an increase in neural and glial progenitors. Taken together, these findings suggest that WDR62 is required for specification of early hPSC-derived neural and glial progenitors.

## Supplementary Material

Supplemental Table 1. Diameter of each neurosphere (NSP) as measured by Image J.
