# Supplementary material for: WDR62 Regulates Early Neural and Glial Progenitor Specification of Human Pluripotent Stem Cells
Source: Stem Cells Int. 2017 Jun 13;2017:7848932. doi: 10.1155/2017/7848932 (PMC5485354; doi:10.1155/2017/7848932)
Supplement: Supplementary file 1 — Supplemental Table 1. Diameter of each neurosphere (NSP) as measured by Image J. [file 7848932.f1.docx]

**Supplemental Table 1.** Diameter of each neurosphere (NSP) as measured by Image J.

| Experiment No. | NSP no.  Dox-treated | Largest width (μm) | NSP no.  Control | Largest width (μm) |
| --- | --- | --- | --- | --- |
| 1 | 1 | 562.837 | 1 | 285.96 |
|  | 2 | 373.609 | 2 | 354.928 |
|  | 3 | 467.7 | 3 | 452.881 |
|  | 4 | 318.65 | 4 | 573.066 |
|  | 5 | 282.906 | 5 | 341.843 |
|  | 6 | 318.281 | 6 | 279.271 |
|  | 7 | 286.109 | 7 | 298.328 |
|  | 8 | 321.758 | 8 | 436.114 |
|  | 9 | 256.659 | 9 | 355.023 |
|  | 10 | 358.669 | 10 | 409.955 |
|  | 11 | 360.58 | 11 | 474.61 |
|  | 12 | 550.52 | 12 | 395.21 |
|  | 13 | 224.718 | 13 | 373.423 |
|  | 14 | 312.103 | 14 | 361.679 |
|  | 15 | 264.131 |  |  |
|  | 16 | 314.093 |  |  |
|  |  |  |  |  |
| 2 | 1 | 398.929 | 1 | 470.083 |
|  | 2 | 419.811 | 2 | 287.121 |
|  | 3 | 444.923 | 3 | 388.225 |
|  |  |  |  |  |
| 3 | 1 | 307.38 | 1 | 542.034 |
|  | 2 | 309.962 | 2 | 466.412 |
|  | 3 | 308.5285 |  |  |
|  |  |  |  |  |
| 4 | 1 | 457.0235 | 1 | 636.307 |
|  | 2 | 369.114 | 2 | 703.218 |
|  | 3 | 627.5485 | 3 | 633.437 |
|  | 4 | 308.1815 | 4 | 630.0975 |
|  | 5 | 377.475 |  |  |
